# Radiotherapy in bone sarcoma: the quest for better treatment option

**DOI:** 10.1186/s12885-023-11232-3

**Published:** 2023-08-11

**Authors:** Marie-Anaïs Locquet, Mehdi Brahmi, Jean-Yves Blay, Aurélie Dutour

**Affiliations:** 1 Cell Death and Pediatric Cancer Team, Cancer Initiation and Tumor Cell Identity Department, INSERM1052, CNRS5286, Cancer Research Center of Lyon, F-69008 Lyon, France; 2 Department of Medical Oncology, Centre Leon Berard, Unicancer Lyon, 69008 Lyon, France; 3 Université Claude Bernard Lyon I, Lyon, France

**Keywords:** Bone sarcoma, Advanced radiotherapy technics, Radioresistance, Radiosensitization

## Abstract

Bone sarcomas are rare tumors representing 0.2% of all cancers. While osteosarcoma and Ewing sarcoma mainly affect children and young adults, chondrosarcoma and chordoma have a preferential incidence in people over the age of 40. Despite this range in populations affected, all bone sarcoma patients require complex transdisciplinary management and share some similarities. The cornerstone of all bone sarcoma treatment is monobloc resection of the tumor with adequate margins in healthy surrounding tissues. Adjuvant chemo- and/or radiotherapy are often included depending on the location of the tumor, quality of resection or presence of metastases. High dose radiotherapy is largely applied to allow better local control in case of incomplete primary tumor resection or for unresectable tumors. With the development of advanced techniques such as proton, carbon ion therapy, radiotherapy is gaining popularity for the treatment of bone sarcomas, enabling the delivery of higher doses of radiation, while sparing surrounding healthy tissues. Nevertheless, bone sarcomas are radioresistant tumors, and some mechanisms involved in this radioresistance have been reported. Hypoxia for instance, can potentially be targeted to improve tumor response to radiotherapy and decrease radiation-induced cellular toxicity. In this review, the benefits and drawbacks of radiotherapy in bone sarcoma will be addressed. Finally, new strategies combining a radiosensitizing agent and radiotherapy and their applicability in bone sarcoma will be presented.

## Introduction

Bone sarcoma are rare tumors accounting for 0.2% of all tumors with an incidence in North America and Europe of 0.75 / 100 000 [1]. Bone sarcoma can be classified according to the age of tumor onset. On the one hand, osteosarcoma (OS) and bone Ewing sarcoma (EWS) mostly affect children and young adults, and on the other hand chondrosarcoma (CHS) and chordoma (CD) occur after the age of 40 [1]. The survival rate of adults with bone sarcoma is low, around 50–60% at 5 years and 30% at 10 years, principally because of the indolent nature of these tumors [2–5]. For localized pediatric bone sarcomas, the 5-year survival rate is around 70% [2–5] and drops to 30% for pediatric bone sarcoma presenting metastases at diagnosis, which occurs in 20–25% of pediatric bone sarcoma [1, 3].

Notwithstanding the age of tumor onset or histological type of sarcoma, the management of all bone sarcoma patients is based on a transdisciplinary approach where surgery, with complete resection of the primary tumor, remains the cornerstone. Indeed, the quality of resection is an essential prognostic factor for all bone sarcomas. Depending on the location of the tumor and the tumoral invasion of peripheral tissues, surgery can be challenging and is not feasible in all cases. Radiotherapy is frequently used to ensure better local control [5–8]. In the case of Ewing sarcoma surgical resection and radiotherapy are both standard options for local control. Conversely, radiations are not applied as first-line treatment for resectable osteosarcoma, chordoma and chondrosarcoma, albeit high doses of radiotherapy are used as adjuvant treatment in case of marginal or incomplete resection, and as definitive local treatment for unresectable tumors [9–11]. Treatment strategy must be adapted according to tumor location, ease of resection and treatment-associated morbidity, as unnecessary high doses of radiation can trigger serious side effects such as neuropathies and fractures [12–14]. Challenging bone sarcoma of the axial skeleton are frequently treated with Intensity Modulated photon Radiation Therapy (IMRT) because of the higher dose applied to the tumor and the sparing of healthy tissues [15]. The development of advanced radiotherapy techniques like carbon-ion, or proton therapy has drastically improved patient care by reducing the exposure of nearby critical organs to radiations and increasing the dose of radiations delivered specifically to the tumor [12–14]. Combined proton and photon radiotherapy is also increasingly used for the treatment of sarcoma of the spine and sacrum and seems to improve local control [12, 16]. Excellent clinical results have been observed for sarcomas of the skull and cervical spine treated with proton therapy [13, 17]. Interesting results have also been reported with heavier particles, such as carbon ion. Access to these advanced RT techniques is increasing in developed countries. Hence, radiotherapy is an important component in bone sarcoma management, and in this review, we will discuss the benefits of radiotherapy for bone sarcoma, the mechanisms involved in tumor radioresistance, and the innovative ways to improve radiotherapy efficacy in these tumors.

### Radiotherapy for bone sarcomas

The place of radiotherapy in the treatment of bone sarcoma has evolved with the development of new types of radiations and new ways to deliver these radiations (Table 1). Nevertheless, this evolution raises the question of choosing the best radiotherapeutic approach for the right tumor depending on patient age, tumor locations, histological subtype, tumor grade, previous treatment. The following paragraphs include an overview of the efficacy of conventional radiotherapy and non-conventional radiotherapy in bone sarcoma.

**Table 1:** Bone sarcomas

*Primary bone tumors are rare, accounting for < 0.2% of malignant neoplasms registered in the EUROCARE (European Cancer Registry-based study on survival and care of cancer patients) database* [1]*.Osteosarcoma and Ewing Sarcoma are the most common malignant bone tumors affecting children and young adults. Osteosarcoma is a complex genomic sarcoma arising mainly in the medulla of long bones while Ewing Sarcoma of the bone (85% of the all the Ewing sarcoma) are high-grade sarcoma arising principally in the diaphysis or metaphysis of the pelvis, femur, or tibia. Osteosarcoma-driven mutations include TP53 and Rb1 while Ewing sarcoma is characterized by the fusion of genes of the FET and ETS family, the most renowned being EWS-FLI1. Chondrosarcoma and chordoma are the most common malignant bone tumors in adults and aging-populations. They affect cartilage cells of the upper arm, pelvis or femur for chondrosarcoma; and cervical, thoracic spine or sacrum for chordoma. Chondrosarcoma and chordoma are thought to arise from the malignant transformation of mesenchymal stem cells and of embryological remnants of the notochord, respectively. Both tumors are highly aggressive locally and present an abundant extracellular matrix. Adult bone sarcoma etiology is not clearly defined and driver mutations are not fully identified even if chondrosarcoma and chordoma initiation seem to be linked to the mutation of IDH genes and T gene, respectively* [1, 18].	

#### Role of radiotherapy in CD and CHS treatment

Over the past few years, more information has become available on the effects of radiotherapy in bone sarcoma patients with unresectable or residual tumors. In this part, we summarize treatment guidelines and present the latest clinical studies evaluating the efficacy of radiotherapy in bone sarcoma (Table 2).

**Table 2:** Radiotherapy in chordoma and chondrosarcoma

Citation		Nb of patients	Information	Treatment background	Overall Survival (OR)	Local control	level of evidence	Type of study	Toxicity
[9]	Dial, et al	1478 chordoma patients, 1401 patients were metastatic	567 skull base, 551 sacral, 360 mobile spine	116 without surgical resection (SR) and radiotherapy (RT), 680 with SR alone, 277 with SR and currative RT, 235 SR and non-currative RT, 59 SR and unknow dose of RT, 111 RT alone	SR + RT improve 5-year OR in patients with positive surgical margin, no effect on patients with negative surgical margin. High-dose RT and new RT model are associated with better outcome compared to standard RT	NA	2b	retrospective analysis	NA
[10]	Krochak et al	38 chondro-sarcoma patients	25 axial: 14 pelvic, 6 limb, 5 spine, 6 head and neck, 7 sternum and rib chondrosarcoma	no patients with complete surgery, 9 patients with chemotherapy combined with radiation	1 death post treatment, 6 survived but very short follow up 8–80 month	17 local failure, 4 distal failure	2b/4	retrospective analysis	NA
[11]	Mc naney et al	20 chondro-sarcoma patients		11 RT alone, 3 with RT and positive SR, 3 RT with chemotherapy, 3 were reccurent tumor after a first surgery	65% OR at 53 month	9 local disease, 8 metastasis	2b/4	retrospective analysis	NA
[12]	Fujiwara et al	48 chordoma, 11 with RT	7 patients with tumor in S1, 7 in S2, 12 in S3, 12 in S4, 6 in S5, 4 in coxcis. Microsa-telite lesion in 3 tumor and vascular invasion in 2 tumors	7 photon, 4 proton therapy	Local Recurrence FreeSurvival (LRFS) 5 years: -SR margin 0 without RT: 50% -SR margin < 1.5 mm without RT 32.8% -SR margin < 1.5 mm combined with RT 85.7% -SR margin > 1.5 mm without RT: 100%	57% of local reccurence without RT, 18% with RT	2b	retrospective analysis	NA
[19]	Catanzano et al	5427 chordoma, 680 RT	Tumor axial and apendicular: -without RT: 44% axial and 56% apendicular -With RT: 78% axial and 22 apendicular	11% metastatic in patients treated with RT vs 4% in patients treated without. 75% with surgery and 44% with positive margin in the RT treated group vs 91% and 12% in the untreated. Chemotherapy in 14% of patients treated with RT vs 5% in the group without 245 patient received conventionnal RT, 245 received advanced	5-year survival rate: -70% in RT treated with a dose > 60 gy, 57 in RT treated with a dosebetween 40 and 60 Gy -78% in advanced chordoma compared to 48% in conventionnal	NA	2b	retrospective analysis	NA
[20]	Zhou et al		clivus and non clivus tumor		-3 year OR: 70% with classical RT, 92% with stereotactic body therapy (SRT), 89% with proton, 93% with carvon ion therapy -5-year OR: 46% with classical RT, 81% with SRT, 78% with proton, 87% with carvon ion -10-year OR: 21%, 40%, 60% and 45% respectively		2a	meta analysis of 25 study (non randomized)	NA
[21]	Gao et al	743 high grade chondrosarcoma	212 axial, 326 extremite, 212 other	SEER stage: 224 localised, 335 regional, 149 distant, 35 unstaged -88% treated with SR, 212 with RT, 172 with RT and SR, 40 with RT alone, 482 with SR alone	5-year OR: 48.5% in patients treated with RT compared to 56% in patients withour RT	NA	2b	retrospective analysis	NA
[22]	Kabolizadeh et al	40 unresected chondrosarcoma	9 cervical, 1 thoracic, 3 lombar, 27 sacral,	all definitive RT	OR at 3 years was 89.1% and 5 years 81.9%	6 local failure, 2 with metastasis. One local and distal failure. 8 metastase (including the 2 with local)	2b	retrospective analysis	acute side effects were grade 1 to 2 radiation-induced dermatitis and pain, nosea and vomiting n = 4, mucositis (n = 5), and diarrhea (n = 5). Long-term toxicities included 10 sacral insufficiency fractures, 2 foot drop, 1 erectile dysfunction, 1 perineal numbness, 2 worsening urinary/fecal inconti- nence, 1 bowel perforation/fistula formation, and 4 grade 2 rectal bleeding
[23]	Palm et al	863 chondrosarcoma, 715 chordoma, non-palliative RT or non-conventionnal RT	various location, skull, vertebra, imb, thorax	NA	Chondrosarcoma DRT: 5-year OR: Proton 75% vs 19.1% for conventional RT. High-dose (> 70 Gy) 40.6% vs 16.9 for low dose. Chondrosarcoma PRT: 5-year OR: proton 97.1% vs 69.4% for conventional RT. High-dose 86.3% vs 69.2% Chordoma DRT: 5-year OR:proton 100% vs 34.1% for conventional RT, and high-dose 79% vs 27%	NA	2b	retrospective	NA
[24]	Lu et al	632 patient, 389 chordoma 243 chondrosarcoma	skull base	NA	OR 1, 5 and 10 years: Chordoma: 100%, 94% and 78% Chondrosarma: 99%, 95% and 79%	LC 1, 5 and 10 years: Chordoma: 99%, 80% and 56%. Chondrosarcoma: 97%, 89% and 88%	2a	systematic meta analyse	Early complications: 24% mucositis, 17% skin irritation, 1% hearing loss Late complications: radiographic brain change 6%, hearing loss 6%, skin reaction 5%
[25]	Imai et al	73 patients, 75 tumor, unresectable chondrosarcoma	26 spinal, 38 pelvic, 11 other	70 conventionnal and 5 dedifferenciated chondrosaroma	5-year OR: 53% Disease free Survival: 34%	5-year Local Control: 53%	2b	retrospective	NA
[26]	wu et al	16 chordoma, 5 chondrosarcoma	19 saccrosigeal, 1 thoracic 1 pelvic. 8 primary and 13 reccurent tumors without metastasis		1-year OR: 100% 2-year OR: 100% Progression-free survival 88.4% and 80.4% respectively	LC 1 and 2 years: 93.8% and 85.2% 5 patients develop lung metastasis	2b	retros	Acute toxicity: 3 grade 1 skin toxicity and 7 grade 1 myelosuppression
[27]	Lockney et al	12 patients included	Chordoma in mobile spine: 6 cervical, 4 thoracic, 2 lumbar	all stereotactic surgery radiation	1 patient with disease progression	Group 1 LC: 80% Group 2 LC (10 month median follow up): 57%	2b	retrospective	4 mucositis, 4 vocal cord paralysis
[28]	Ryugi Nakamura et al	1 patient	Pulmonary metastases for extraskeletal mucinous chondrosarcoma	stereotactic body radiation therapy	healthy for another 4 years	NS	5	case report	pneumotitis
[29]	Vasudevan et al	20 patients	16 chordoma and 4 chondrosarcoma (4 recurrences)	Fractionnated Stereotactic radiotherapy peri-operatively	28-month OR: 90%	LC: 90%	2b	retrospective	9 patients with grade 1–3 acute toxicity, 2 patients with grade 4, 5 toxicity

In chondrosarcoma, radiotherapy can be considered for unresectable disease (primary or recurrent), after incomplete surgery and for symptom palliation. High-dose RT is currently recommended for patients with skull base chondrosarcoma and for inoperable, locally advanced, and metastatic high-grade chondrosarcomas with a poor prognosis. For chordoma, en bloc R0 resection is the recommended treatment for primary localized disease when feasible and sequelae are accepted by the patient. If these conditions are not met, RT alone without debulking is an alternative. For skull base and upper cervical tract chordoma, resection with negative margins can rarely be done, and microscopically-positive margins should be the goal of surgery. Adjuvant RT should be considered for skull base and cervical spine chordomas, and for sacral and mobile spine chordomas with R1 resection margins.

A few historical retrospective studies have been conducted to determine whether chordoma and chondrosarcoma patients could benefit from peri-operative radiotherapy. Two major retrospective studies have evaluated the role of radiotherapy in chordoma, comparing surgery alone vs surgery and conventional radiotherapy in 1478 and 5427 chordoma patients, respectively (level of evidence 2b) [9, 19]. Both studies concluded that radiotherapy peri-operatively improves patient local control when surgery with positive margins are performed. High-dose RT is also associated with better outcome [9, 19]. The same observation has be made in a retrospective study of 743 high-grade chondrosarcoma defining radiotherapy as an independent protective factor (level of evidence 2b) [21].

Different advanced radiotherapeutic techniques have been developed in the last few decades (Table 3, see Table 2 [9–35]). First, the use of proton therapy is associated with better outcome than conventional radiotherapy in both chordoma and chondrosarcoma [9, 19, 21, 23]. The administration of proton and photon therapy post-operatively tend to be more efficient with a 5-year local control rate of 85.4% in CD, while it does not exceed 74% when combining surgery and photon radiotherapy alone [22]. When radiotherapy is administered as a single treatment (*e.g.* in unresectable tumors), proton therapy is a better option than conventional radiotherapy for both CHS and CD, resulting in a 5-year overall survival of 75% for CHS and 100% for CD, whereas the 5-year overall survival is only 19.1% for CHS and 34.1% for CD for conventional radiotherapy [23]. In skull base chordoma and chondrosarcoma, which are particularly difficult to handle surgically due to their proximity to vital structures, carbon ion radiotherapy administered peri-operatively has shown promising results with a 5-year local control of 80% and 89% in CD and CHS, respectively [20]. Stereotactic Radiation Therapy (SRT) has also been used in both chordoma and chondrosarcoma, and retrospective studies reported different results, with local control rates varying between 57% at 10 months and 90% at 28 months [28, 29].

**Table 3:** Radiotherapy principles

*Radiotherapy is one of the most widely used therapies for tumors. Radiation is defined as “ionizing” if its energy load is enough to ionize a molecule of water (> 10 eV). There are two categories of ionizing radiations: particle beams (protons, neutrons, ions, α-particles) and photons radiations (X-rays, γ-rays). Ionizing radiations are characterized by their capacity to ionize a tissue, or Linear Energy Transfer (LET). Particle beams have high LET and photon radiations have low LET. External beams are generally used to deliver the maximum dose of radiation to the tumor and to spare surrounding healthy tissues. Different strategies of radiation delivery can be adopted depending on the patient and the type of tumor: 3D conformational radiation is adapted to the shape of the tumor by delivering beams from different directions. More recently, advances in imaging promoted the use of Intensity Modulated Radiation Therapy (IMRT). IMRT uses smaller beams with different intensities to deliver different doses of radiation to certain areas of the tumor. For example, higher doses can be delivered to hypoxic areas which are usually more radioresistant, while sparing healthy tissues near the tumor. Variable radiation intensity is generated across each beam, in contrast to the uniform intensity used in other RT technics. Stereotactic Body Radiation Therapy is a technique that uses precise imaging in conjunction with high-intensity radiations beams to deliver high radiation doses to tumors in three to five treatments. Extracorporeal radiation can also be used in the treatment of bone sarcoma and consists in excising the tumor bearing segment of bone, irradiate the tumor and reimplant it back into the body.*	

Chondrosarcoma and chordoma have a very low incidence, thus international clinical trials uniting bone sarcoma centers worldwide are ongoing to determine the best therapeutic option depending on the type of the tumor, its localization (NCT05033288, NCT01182779) and its resectability (NCT02986516).

#### Role of radiotherapy in the treatment of Ewing sarcoma and osteosarcoma

Radiotherapy may be considered in osteosarcoma patients with unresectable tumors, primary tumors where surgery would be unacceptably morbid, or as adjuvant treatment of tumors at high risk of local recurrence and with limited option for further surgery. For patients with bone Ewing sarcoma, RT with definitive intent alone should be used instead of surgery if complete surgical excision is not possible and in cases with challenging local sites such as axial or spinal tumors, where surgery will be unacceptably morbid. Adjuvant RT (45–60 Gy) significantly reduces Local Recurrence in patients with large tumors (> 200 ml), poor histological response or inadequate surgical margins and should be recommended in these circumstances [IV, B].

In addition, adjuvant RT should be considered in patients with non-sacral pelvic Ewing Sarcoma regardless of surgical margins, tumor volume or histological response, as this was shown to provide superior local control and survival outcome compared with surgery alone.

Several studies aimed at determining the best use of radiotherapy for EWS patients comparing radiotherapy alone with i) surgery alone, ii) post-operative RT, or iii) polychemotherapy (see Table 4). In a retrospective study (INT0091, INT0154, AEWS0031), radiotherapy alone increased the rate of local relapse compared to surgery alone in EWS patients with localized tumors [31].However, no difference was observed in the overall survival and overall disease control between those two treatments [30]. For patients with extremity and pelvic tumors, surgery clearly improved local control compared to definitive radiotherapy (local relapse rates 3.7% and 3.9% vs 14.8 and 22.4%, respectively) [30]. For other tumor locations, no difference was detected between the different treatment groups. Of note, in this study, patients treated with surgery had favorable prognostic factors such as a younger age or tumors of the extremities, and most of the patients were treated with older techniques of radiotherapy. Another study compared the same treatment options (surgery vs radiotherapy vs combined treatment) in metastatic EWS. The combination of surgery and radiotherapy improved the local control of metastatic tumors compared to surgery or radiotherapy alone (EFS at 3 years: RT: 0.35, surgery: 0.35, combination: 0.56) [31].

**Table 4:** Radiotherapy in Ewing sarcoma and Osteosarcoma

Citation		Nb of patients	Information	Treatment background	Overall Survival (OR)	Local control	level of evidence	Type of study	Toxicity
[32]	Delaney et al	41 osteosarcoma	unresected or incompletely resected. 27 primary disease, 10 local recurrence and 4 metastatic disease	photon and/or proton beam therapy	NA	LC at 5 years: 78.4% (total resection); 77.8% (subtotal resection), 40% (biopsy only)			NA
[30]	Dubois et al	465 bone Ewing sarcoma	All non-cranial: 124 distal extremity, 123 proximal, 98 pelvic, 95 chest wall, 25 spine	RT alone for 121 patients, SR alone for 241, RT and SR combined for 103	Compared with surgery, radiation had a higher risk of local failure (HR, 2.41; 95% CI, 1.24–4.68. No difference in event-free survival (EFS)		2a	retrospective of 3 combined study	NA
[31]	Haeusler et al	120 Ewing sarcoma	For primary tumor, 26 patients SR alone, 21 SR and RT, 40 RT alone. For metastasis 6 SR, 9 SR and RT, 33 RT. All patients received chemotherapy. Almost all patients presented metastasis (82.2% bone, 43% Bone Marrow, 22% lymph node)	Forty-seven (39%) patients had local treatment of both the primary tumor and metastases, 41 (34%) patients of either the primary tumor or metasta- ses, and 32 (27%) received no local therapy. Primary tumor location: 82 central, 34 peripheral, 4 unknown		3-year EFS was 25% with SR, 47% with SR and RT, 23% for RT, and 13% when no local therapy was admin- istered	2b	retrospective	NA
[33]	Brown et al	Stereotactic body radiotherapy: 14 patients: 9 osteosarcoma and 5 ewing	13 metastatic patients, 27 lesions treated (19 osteosarcoma and 8 ewing)	21 bone lesions and 6 pulmonary 1/3 of the case were co treated with chemotherapy			4	descriptive report of faisability	Two grade 2 and one grade 3 complication: myonecrosis, avascular necrosis with pathologic fracture, and sacral plexopathy
[34]	Mohamad et al	26 unresectable pediatric osteosarcoma. Carbon ion radioterapy	24 pelvic, 1 mediastinal and 1 paravertebral	22 primary, 1 local reccurent, 3 meta	OR: 50% and 41.7% at 3 and 5 years	LC 69.9% and 62.5% at 3 and 5 years Progression-free survival was 34.6% at 3 and 5 years	2b	retrospecive	4 grade 3–4 CIRT-related late toxicities, 1 case of bone fracture and no treatment-related mortalities
[35]	Seidensal et al	Combined ion-beam radiotherapy combined with carbon ion or proton	20 patients with primary (N = 18), metastatic (N = 3), or recurrent (N = 2) tumor. Inoperable pelvic (70%) or craniofacial (30%) osteosarcoma treated with protons up to 54 Gy (RBE) and a carbon ion boost of 18 Gy (RBE)	3 surgery before treatment, all r2. All patient with chemotherapy treatments. Three patients with metastatic disease (15%) received radiotherapy of their primary tumor only but not for the metas- tases	OR 75% at one year and 68% at two	Local progression-free survival 73% at 1 year and 55% at two Distal progression-free survival: 74% at 1 and 65% at two years. Global progression-free survival 60% and 45% respectively	2b	retrospective	No acute toxicities > grade III were observed. One case of secondary acute myeloid leukemia (AML) seven months after CIBRT for recurrent disease and one case of hearing loss

Stereotactic Body Radiation Therapy (SBRT) (Table 1) uses several radiation beams of various intensities targeting the tumor from different angles and is considered an effective strategy for metastatic EWS and OS [33]. SBRT used to control pulmonary metastases was reported to lead to a 2-year local control of 60% in 13 metastatic patients (IV) [33]. In osteosarcoma, the local control at 5 years was shown to range between 68 and 72% with conventionally fractionated proton RT doses of 68-70 Gy (1.8-2 Gy per day) in a retrospective study including 41 OS unresected or incompletely resected [36]. Carbon ion radiotherapy was effectively used in the treatment of unresectable pediatric osteosarcoma, with a local tumor control of 62% at 5 years in a retrospective study [34]. More recently, Combined Ion Beam Radiotherapy with protons and carbon ions in a multimodal treatment strategy of inoperable osteosarcoma was evaluated. Results showed an overall survival and a progression-free survival of 68% and 45%, respectively (2b). These results are particularly promising in craniofacial osteosarcoma [35]. Recently, a randomized controlled phase III study evaluated the efficacy of carbon ions, photon, and proton therapy in chordoma and chondrosarcoma (except skull-based tumors). This study will be extremely valuable in determining the benefits of using carbon ion radiotherapy as it is a prospective study and it compares the effects to a reference treatment [37].

Even though chemotherapy is a preferred treatment choice, RT plays a primordial role in the treatment of bone sarcomas. The development of new techniques makes RT an approach of interest for the treatment of incompletely or unresectable tumors, for tumors localized near critical structures, and for metastases. These new radiotherapies can lead to a better management of sarcoma patients who have an unfavorable prognosis and limited treatment options. With great advances in the development of targeted therapies, moving on to personalized combination approaches able to enhance the efficacy of radiotherapy, may be a promising strategy. To achieve this goal, a better understanding of radiotherapy mechanisms of action is necessary.

### Potential target for combination with radiotherapy in bone sarcomas

Radiotherapy is currently focused on the precise delivery of high doses of radiation within the tumor bulk, sparing surrounding healthy tissues. However, the development of targeted therapy arguably has the potential to enhance radiotherapy efficacy. The possibility to molecularly profile tumors at diagnosis, together with improvements in radiotherapy could potentially pave the way for a more personalized approach to bone sarcoma treatment. Several key molecular pathways could theoretically enhance the therapeutic effect of radiation. In addition, it is important to determine the timing for combining molecular targeted therapy with radiation, as it could greatly affect the outcome depending on which pathway is being inhibited.

**Fig. 1:**
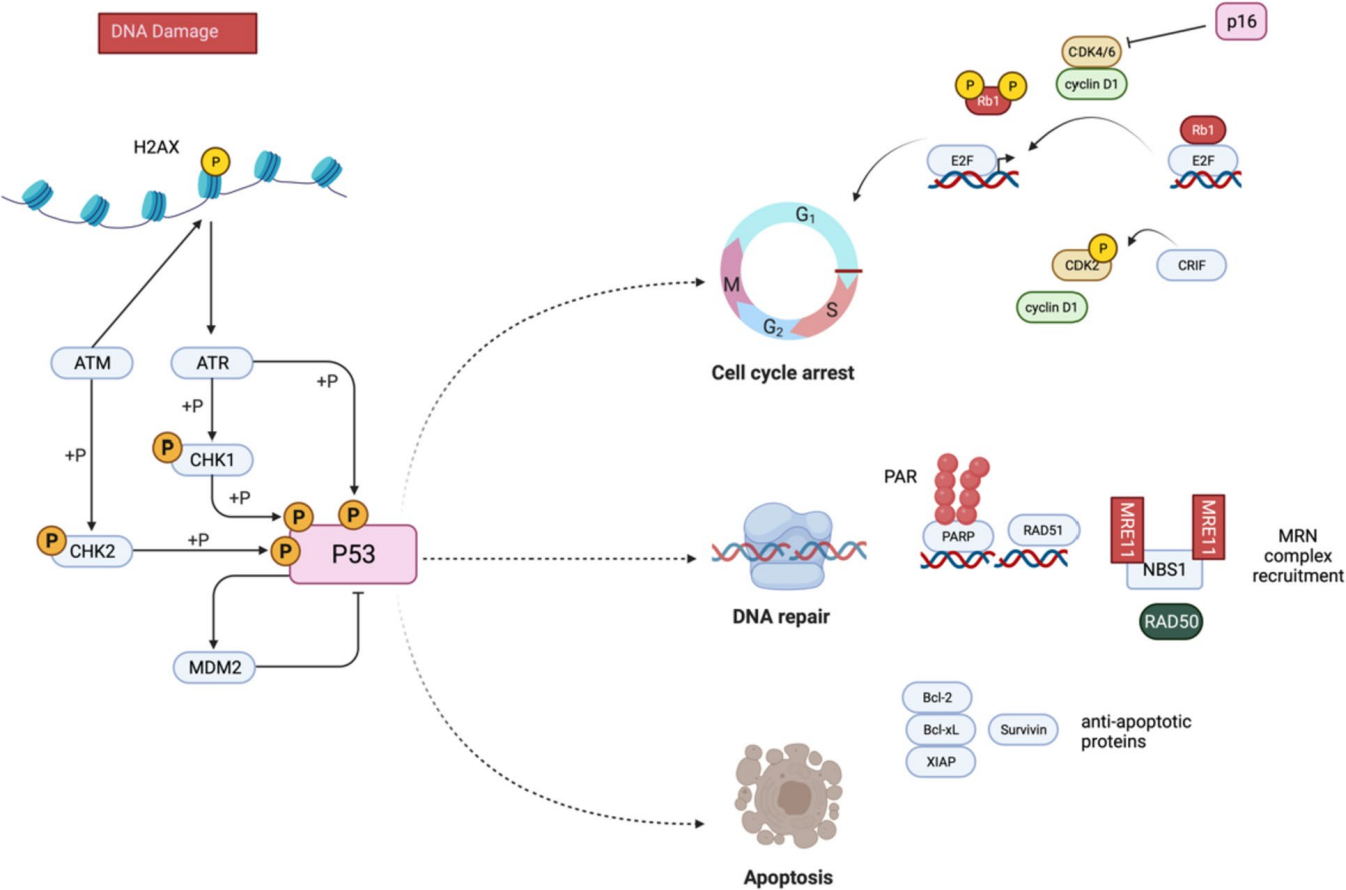
Schematic representation of the major known actors involved in radioresistance of bone sarcomas- when a damage occurs in DNA, ATM and ATR kinases are recruited and activate checkpoint kinases 1 and 2, leading to cell cycle arrest and to the recruitment of diverse effectors of DNA repair, such as the complex MRN PARP, RAD51, NBS1, RAD50. Diverse alterations in cell cycle proteins including p16 and CRIF, and in DNA repair proteins enhance bone sarcoma radioresistance. The accumulation of DNA damage is generally leads to cell death. However, bone sarcoma cells present defects in this pathway too, leading to cell survival after radiotherapy. Created using BioRender.com

To determine which potential pathway could be a promising target in bone sarcomas, it is first necessary to review the radiation process and its consequences at the cellular and molecular levels. This paragraph summarizes, in chronological order, the principal steps and actors involved in the cellular response to radiotherapy (Fig. 1).

Irrespective of the type of radiations used (*e.g.* X-rays, Proton, carbon ion), ionizing radiation affects all cellular compartments and their main target is DNA. Under ionizing radiations, micro-deposits of energy are generated in the nucleus near DNA. This accumulation of energy destabilizes and causes damage to the DNA structure. Moreover, by ionizing water molecules, a phenomenon known as water radiolysis, radiation triggers the formation of Reactive Oxygen Species (ROS) that lead to further DNA damage. DNA damage caused directly or indirectly by radiations, includes DNA oxidation, loss of a base, single-strand break and double-strand breaks [38, 39]. Double-Strand Breaks (DSB) are considered the most lethal type of lesions and are induced at a higher level by proton rather than photon therapy [40]. Each type of damage is recognized and corrected by specific repair mechanisms, each acting with a different degree of precision and speed (Table 5).

**Table 5:** Biological differences between photons, protons, and carbon ions

X*-rays have no mass and interact weakly with matter, depositing energy along their entire path until they exit the body. The highest doses are recorded just below the skin, and deep-seated tumors can be treated by focusing beams from many different angles. The energy deposited by X-rays is diffuse, hence X-ray radiation is characterized by low linear energy transfer (LET). Protons and carbon ions are charged particles with mass that have the important property of depositing low amounts of LET energy when traveling at high speed through tissue. Collision of these particles with tissue causes the particles to slow down and eventually stop, and they deposit the bulk of their energy at the very end of their path (Bragg Peak). Because no energy is delivered beyond the particle stopping point, normal tissue situated beyond the tumor receives almost no dose. While low LET radiations produce diffuse ionizations along their tracks, high LET radiations cause dense ionizations that create clustered DNA damage that is less easily repaired by tumor cells. This is reflected in the greater tumor cell killing per unit of dose of high LET radiations (carbon ions) compared to low LET radiations (photons, protons). This difference is termed Relative Biological Effectiveness.*	

The response of a cancer cell to an ionizing radiation can be divided into several steps, from the recognition of the damage to the induction of cell death. At each step, bone sarcoma cells can have properties allowing them to counteract radiation-induced cell death, representing potential targets for combination therapy (Tables 6 and 7). Most of the studies on the biological effects of radiotherapy in bone sarcoma focus on X-rays or γ-rays, which will be presented in the next paragraph, and since very few studies (only 2 studies) deal with protons or carbon ions these will be presented when necessary.

**Table 6:** TP53 mutations in bone sarcomas

**Sarcoma**	**Overall TP53 mutation rate**	**TP53 mutations**	**Other mutations affecting TP53**
OS	80%	*TP53* intron rearrangements	*MDM2/MDM4* gene amplification
EWS	10%	*C176F* and *R273X*	Inhibition of WT *TP53* by EWS-FL1 fusion protein
CHS	20%	*TP53* intron rearrangements	*MDM2* amplification Alterations in the *TP53* pathway
CD	1–2%	*TP53* missense mutations	/

**Table 7:** Combination of radiotherapy and pharmacological inhibition of targets in bone sarcoma

Drug	Drug target	RT technic used	Models	Combination effects	Citation
**Osteosarcoma**					
Zoledronic acid	Osteoclasts	γ radiation	KHOS/NP, U-2, MG63, HOS OS cells	Increased cell death, increased levels of ROS, increased DNA damage, decreased proliferation	[41]
Sulforaphane	Multiple targets: survivin, NFKB, Bcl-2, VEGF, MMP-2	X-rays	LM8 murine OS cells	Cell cycle arrest, increased DNA damage, increased apoptosis, decreased cell proliferation	[42]
Ginseng polysaccharide	Multiple targets	γ radiation	MG63 cell line	Decreased cell viability, increased apoptosis and autophagy,	[43]
BI6727, GSK461364	PLK1, key regulator of mitosis	X-rays	HOS and MG63	Cell growth arrest, apoptosis induction	[44]
KU60648	DNA-PKcs, serin/threonine kinase, sensor of DNA damage	γ radiation	143B, U2OS, Saos-2, Hos	Altered cell cycle distribution, increased DNA damage, decreased survival fraction	[45]
SAHA	HDAC, histone deacetylase	X-rays	KHOS-24OS, SAOS2 cell lines, xenogrqfted mice	Increased cell death	[46]
Hydrogen peroxide	ROS induction	X-rays	HS-Os-1 cell line	Oxidative DNA damage induction	[47]
Valproic acid	HDAC, histone deacetylase	X-rays	U2OS cells	Decreased cell survival, increased chromosomal abberations	[48]
SAHA, M344, PTACH	HDAC	Proton therapy	U2OS	Decreased survival fraction, increased DNA damages	[49]
SAHA, M344, valproate	HDAC	X-rays	KHOS-24OS, SAOS2	Decreased survival, cell cycle arrest, enhanced apoptosis	[50]
Demethylating agent 5-Aza-CdR	Methylation, regulation of genic expression	X-rays	SaOS, HOS, U2OS	Enhanced apoptosis, arrest in G2/M	[51]
Berberine, isoquinoline alkaloid	Multiple targets	γ radiation	MG63	Increased cell death, induced cell cycle arrest in G2/M, induced apoptosis	[52]
DTCM-g	Activator Protein 1	X-rays	HOS MG63	Decreased cell proliferation	[53]
BI2536	PLK1, key regualtor of mitosis	X-rays	U2OS	Cell cycle arrest, increased cell death	[54]
Wortmannin	PI3K, proliferation and survival	X-rays	MG-63	Decreased cell survival fraction, decreased DNA repair	[55]
**Ewing sarcoma**					
Mithramycin	Inhibitor of transcription	X-rays	4 EWS:Fli1 + and 3 EWS:Fli- cells in vitro and in vivo	Reduced tumor growth in vivo, increased apoptosis	[56]
Olaparib	PARP-1	γ radiation	RD-ES, SK-N-MC EWS cell lines + tumor xenografts SK-N-MC	Decreased proliferation, increased cell death	[57]
Curcumin	Multiple targets	γ radiation	SK-N-MC cell lines	Increased apoptosis and DNA fragmentation, increased cytotoxicity	[58]
Taxol	Multiple targets	X-rays	Cell line HTB-166	Blockade in G2/M, decreased colony formation rate	[59]
**Chondrosarcoma**					
Olaparib	PARP	X-rays, proton, hadron therapy	CHS2879 cell line	Decreased cell survival, decreased proliferation	[60]
Disulfiram + copper	ALDH1A1	X-rays	SW1353 and CS1 cell lines, Orthotopic CHS model,	Decreased survival, increased apoptosis, decreased colonies, decreased cancer stem cells	[61]
**Chordoma**					
Hyperthemia		X-rays	U-CH2 and MUG-Chor1 cell lines	Reduced colony formation	[62]
Ribavirin	Anti-viral drug	X-rays	U-CH1 cell line in vitro and in vivo	Decreased cell growth in vitro and in vivo	[63]
LB100	Protein Phosphatase 2A	X-rays	U-CH1, JHC7, UM-ChOR1 in vitro + in vivo	Accumulation in G2/M, growth inhibition, in vivo tumor growth delay	[64]
DIMATE	ALDH1, ALDH3	X-rays	U-CH1, U-CH12, CH22 3D	Decreased proliferation, decreased colony formation, increased cell death	[65]

#### DNA damage recognition

DNA damage is first recognized by 2 enzymes: Ataxia Telengiectasie Mutated (ATM) and Ataxia Telengiectasie RAD3-related (ATR). ATM recognizes double-strand breaks, while ATR can detect single-strand breaks and replication fork alterations. After the recognition of a DSB, ATM phosphorylates the histone H2AX (yH2AX), involved in stabilizing DNA extremities and in the recruitment of DNA repair complexes. ATM and ATR also phosphorylate the checkpoint kinases 1 and 2 (CHK1 and CHK2), leading to cell cycle arrest. ATR can phosphorylate many other substrates including Replication Fork components: MCM (MiniChromosome Maintenance) proteins, Rpa (Replication Protein A), polymerase, PCNA (Proliferating Cell Nuclear Antigen), and Claspin (Mrc1) [66, 67]. Cancer cells can resist radiation by increasing their efficiency in DNA repair through the increased expression of proteins involved in DNA damage recognition and repair, including ATM and ATR. A correlation was shown between radioresistance levels and the expression of 7 proteins involved in the DSB DNA repair machinery in 5 sarcoma cell lines, including one OS cell line. ATM, ATR and NBS (Nijmegen breakage syndrome protein 1), proteins involved in DNA damage recognition presented the strongest correlation [68]. In CD, an increased expression of ATM, ATR and yH2AX was observed in 26 patient samples in comparison with surrounding healthy tissue. However, this observation has not been directly correlated to the level of radioresistance of CD [69, 70]. Drugs targeting both ATR and ATM are already approved by the FDA and in clinical trials in other cancers (Bay1895344, NCT03188965; AZD1390, NCT03423628).

Once activated, ATM, CHK1 and CHK2 phosphorylate p53, the most studied tumor suppressing protein. P53 is the protein the most often mutated in all cancers and plays major roles in genomic stability, cell cycle regulation, cell death induction and in radioresistance.

#### P53 activation

P53 is a transcription factor that is stabilized following radiation and induces transcription of genes associated with cell cycle arrest, apoptosis, and metabolism, thereby functioning as a tumor suppressor [71]. Mutations affecting the normal functions of p53 are found in 80% of OS, 20% of CHS, and 10% of EWS (Table 6) [72]. Typically, the majority of TP53 mutations are missense mutations in its DNA binding domain, preventing TP53 from inducing transcription of its target genes and thus causing the loss of its tumor suppressive function [71]. In OS and CHS TP53 functions can also be altered indirectly through the amplification of Murine Double Minute 2 (MDM2) that results in P53 degradation. Recent results have demonstrated that TP53 mutations are associated with a radioresistant phenotype and poor survival in EWS patients [73]. TP53 is rarely mutated in CD; a whole genome sequencing study conducted on 63 CD samples revealed that only one sample carried a p53 mutation [74]. However, an increased expression of p53 was observed in 9/10 patients presenting relapsed tumors compared to patients with a stabilized disease. Thus, in chordoma overexpression of TP53 is correlated with tumor relapse and is a poor prognostic factor [75, 76]. Other studies are needed to understand the role of p53 in CD radioresistance.

If TP53 involvement in radioresistance is quite clear, further molecular studies are needed to precisely determine the underlying mechanisms of p53-driven radioresistance in bone sarcomas in terms of effectors and functions. In addition, although multiple p53 reactivators have been developed, only two drugs have entered clinical trials, APR-246 and COTI-2, currently making p53 hardly targetable.

#### Cell cycle arrest

Cell cycle regulation is a critical biological function involved in response to radiation. Arresting cell cycle progression is an essential step to enabling the recruitment of DNA repair machinery when DNA damage is caused by radiations. Several major actors of cell cycle regulation are involved in bone sarcoma radioresistance (Fig. 1). The gene Cyclin Dependent Kinase Inhibitor 2A (CDKN2A) encodes the P16 protein that inhibits Cyclin Dependent Kinases 4 and 6 (CDK4/6), inducing cell cycle arrest in G1 phase [77]. CDK4/6 usually bind to cyclin D1 and phosphorylate the tumor suppressor protein Rb1. The phosphorylation of Rb1 prevents its binding to the protein E2F, which in turn activates the transcription of genes allowing entry into the S phase [78].

The CDKN2A locus, is frequently deleted in bone sarcomas [74, 79–81]. The absence of p16 allows CDK4/6 activation and entry into the S phase of the cell cycle and could represent an advantage for cancer cells in response to radiation. These alterations could explain their low sensitivity to radiation. Pre-clinical studies refer to the synergistic effect of CDK4/6 inhibitors-radiotherapy combination. For instance, different clinical studies are ongoing in other cancers to determine the efficacy of combining radiation therapy and Palbociclib in breast cancer patients (NCT03691493, NCT03870919) and in locally advanced squamous cell carcinoma (NCT03024489). Further studies need to be done to determine the therapeutic potential of CDK4/6 inhibition in combination with radiotherapy.

Another protein involved in sarcoma radioresistance is CRIF, a protein regulating cell cycle. This protein phosphorylates CDK2, inducing cell cycle arrest and promoting DNA repair [82], a strong expression of CRIF has been detected in OS patient samples. CRIF inhibition by siRNA in both OS cell lines and OS xenografts was shown to increase sensitivity to irradiation, delay DNA damage repair, inactivate G1/S checkpoint, induce mitochondrial dysfunction and tumor regression in vivo [82]. Other strategies aimed at inhibiting cell cycle to reinduce radiosensitivity. In OS, the inhibition of PLK1 [5, 54], WEE1 [83], or PI3K [55] combined to radiotherapy generated cell growth arrest and cell death through mitotic catastrophe. Other studies are urgently needed to decipher the therapeutic potential of cell cycle gene alterations.

Once DNA damage is recognized and the cell cycle is arrested, the next step in cellular response to radiation is DNA repair.

#### DNA damage repair (DDR)

DNA repair involves a complex machinery and is orchestrated by numerous actors. Here, we will present the major DNA repair actors involved in the response of bone sarcoma to radiation-induced DSBs. For DSB DNA repair, two major pathways are activated: homologous recombination (HR) and non-homologous end joining (NHEJ).

NHEJ occurs during the G1 phase; it binds broken DNA extremities together leading to an increased number of errors. NHEJ initially recognizes DNA damage through a heterodimer Ku70-Ku80. This complex block 5’ DNA extremity and maintains DNA extremities close to each other to allow their binding. This complex also activates the protein 53BP1, which protects DNA extremities from more damage. γH2AX phosphorylation by ATM is also involved in stabilizing DNA extremities. The final steps following assembly of the repair machinery involve binding of DNA extremities by ligases (LIG4, XRCC4, and XLF) [84].

Homologous recombination (HR) only takes place in late S and G2 phases of the cell cycle, as this DNA repair mode is based on the use of the sister chromatid to synthesize an identical DNA strand. This reparation system is more precise than NHEJ. Here, The DNA DSB is recognized by the MRN complex composed of 3 proteins (MRE11, RAD50, NBS1), which initiate resection of DNA extremities in collaboration with CTIP. A loop with the sister chromatid is then formed and a DNA polymerase replicates DNA and ligases bind DNA to the strand break [84, 85]. Certain strategies aim at inhibiting DNA repair to induce cell death such as RAD51 inhibition, a recombinase involved in the DDR machinery. In OS and CD, the inhibition of RAD51, combined with radiations lead to a decreased cell proliferation and an increased apoptosis [86, 87]. In CHS and EWS, the PARP1 inhibitor Olaparib in combination with radiations was reported to decrease cell survival and clonogenic capacities [57, 60].

In this system, PARP-1 is rapidly recruited and activated by DNA DSBs. Upon activation, PARP-1 synthesizes a structurally complex polymer composed of ADP-ribose units that facilitate local chromatin relaxation and the recruitment of DNA repair factors [57]. In both CHS and EWS, PARP-1 seems to play a role in radioresistance. In 2 EWS cell lines, the combination of the PARP-1 inhibitor Olaparib and radiation therapy was more effective than radiotherapy or Olaparib alone. This combination induced a 4-fold increase in apoptosis in comparison with both treatments alone and lead to increased and sustained DNA damage in EWS cell lines. Moreover, in in vivo xenografts models of EWS, the combination of Olaparib and radiation therapy stopped tumor progression [57]. In the CHS cell line CH2879, Olaparib enhanced the efficacy of radiation by 1.3-fold for X rays, 1.8-fold for protons and 1.5-fold for carbon ions [60]. In a study of 11 advanced CD, a mutational signature associated with HR deficiency was found in 72.7% of samples, co-occurring with genomic instability. The use of Olaparib led to prolonged survival in a patient with refractory advanced CD [70]. Olaparib is currently being dose escalated in combination with radical (chemo-)radiotherapy regimens for non-small cell lung cancer (NSCLC), breast cancer and head and neck squamous cell carcinoma (HNSCC) in three parallel single institution phase 1 trials (Study protocols of three parallel phase 1 trials combining radical radiotherapy with the PARP inhibitor Olaparib).

After exposure to radiation, cells normally accumulate DNA damage that cannot be repaired fast enough and with enough precision for the cell to reenter the cell cycle. Proteins involved in genomic stability such as p53 then trigger cell death. However, sarcoma cells often lack the proteins supposed to control genomic integrity and present defects in cell death pathways [88].

#### Cell death

In response to DNA damage, apoptosis can be induced by different ways: i) activation of p53 or ii) accumulation of ROS. TP53 can directly promote cell death after DNA damage or after incomplete repair of DNA damage [89]. This is mediated through the activation of pro-apoptotic proteins, such as Tumor Necrosis factor Receptor superfamily (TNFR), triggering the extrinsic apoptosis pathway [90].

ROS accumulation can also induce cell death through the loss of mitochondrial membrane potential, leading to the release of cytochrome c. Moreover, ROS cause lipid damage, which activates sphyngomyelinase and induces the production and release of ceramide that in turn can activate caspases 1, 3 and 6, leading to cell death [91, 92].

An incomplete DNA repair can also induce a mitotic catastrophe, during which an abnormal chromosomal condensation occurs and the cell enters in mitosis before the end of S and G2 phases of the cell cycle [93].

Few studies have focused on the involvement of cell death defects in the response of bone sarcoma to radiotherapy. In CHS, the anti-apoptotic proteins Bcl-2, Bcl-xL and XIAP were found to be overexpressed in 2 CHS cell lines in comparison with 2 normal chondrocytes cell lines. When the expression of these anti-apoptotic proteins was inhibited by siRNA, a 10-fold increase in radiosensitivity was observed in CHS cell lines [94]. In EWS cell lines, an exposure to 2 to 10 Gy X-rays was reported to increase the expression of the anti-apoptotic protein survivin in a dose-dependent manner. Survivin inhibition by siRNA doubles apoptotic cell death [95, 96]. Several BH3 mimetics are currently used in the clinic, for example Venetoclax is approved for routine clinical practice in chronic lymphocytic leukemia (CLL) and acute myeloid leukemia (AML). To our knowledge, BH3-mimetics have not yet been combined to radiotherapy in patients.

Bone sarcomas arise in a particular environment (*i.e.* the bone or cartilage) and one of the characteristics of this environment is its hypoxic content that plays a role in resistance to conventional radiotherapy. Other factors of the tumor microenvironment, like the presence of immune cells or the extracellular matrix are likely involved in bone sarcoma radioresistance but studies regarding these are lacking.

#### Hypoxia

Hypoxia is a common feature of solid tumors, resulting from the imbalance between oxygen availability and consumption, and is defined as one of the most important causes of radiotherapy failure [97]. In bone sarcoma, the presence of hypoxic zones is correlated with tumor relapse, metastases and resistance to treatments [98–102]. These hypoxic zones are also predictive of poor tumor response to conventional radiotherapy. Different mechanisms have been suggested to explain the link between bone sarcoma, radioresistance and hypoxia. Evidence suggests that hypoxia inhibits the indirect effects of radiotherapy driven by the accumulation of ROS, creating more damage in cells which finally undergo cell death. The first mechanism proposed for hypoxia-induced radioresistance is the acceleration of ROS clearance. In a study including 35 OS and 20 EWS samples, it was shown that radiotherapy does not affect oxidative stress levels. However, it is known that radiotherapy induces ROS production which should increase oxidative stress. Hence, if oxidative stress levels remain constant, this implies that ROS clearance in the tumor cells is accelerated. The activation of autophagy and increased antioxidant metabolism are two hypotheses which can explain how sarcoma cells can accelerate ROS clearance. Indeed, it was demonstrated in OS that hypoxia confers cells resistance to radiation through activated autophagy to accelerate the clearance of cellular ROS products [103]. The increased antioxidant metabolism, mediated by the increase in two antioxidant enzymes, namely Aldehyde dehydrogenase (ALDH) 1 and 3, was shown in CD in an in vitro study. In this study, the pharmacological inhibition of the ALDH1 and 3 restored radiosensitivity to CD spheroid models in vitro [65]. Hypoxia-induced conventional radioresistance can potentially be counteracted by the addition of proton therapy, which has a higher efficacy in hypoxic zones (NCT02802969).

#### Other potential therapeutic targets with pre-clinical efficacy

Inhibition of histone deacetylases or demethylating agents has proven to be effective in combination with radiation, particularly in OS. Indeed, Histone DeACetylase (HDAC) inhibitors in combination with radiation was reported to increase cell death and DNA damages in several OS cell lines [46–51]. In CD and CHS, strategies targeting cancer-initiating cells (CIC) have been tested. One study highlighted that the use of disulfiram, an FDA-approved anti-alcoholism drug, complexed with Cu can radiosensitize CHS CIC. Indeed, the addition of DSF/Cu to a CHS cell line and a CD cell line decreased the clonogenicity of cells, while increasing apoptosis. Moreover, in an orthotopic model of CHS, the combination of DSF/Cu and radiation induced a strong decrease in tumor growth [61]. Similar results were obtained in CD, where the inhibition of ALDH1 and 3, proteins overactivated in CIC, radiosensitized 3D culture of CD cell lines [65]. Efforts need to be made to evaluate the potential of other radiosensitizing strategies. To do this, genetic inhibition of targets in combination with radiotherapy have been tested (Table 8).

**Table 8:** Combination of radiotherapy and genetic inhibition of targets in bone sarcoma

Target	Method of inhibition	Models	Results	Citation
**Osteosarcoma**				
CRIF1	Knock down	U2OS cells + xenografts	Increased sensitivity to irradiation, delayed DDR, inactivated G1/S checkpoint, mitochondrial dysfunction. Tumor regression in vivo	[82]
miR-513a-5p	Treatment with miR-513-5p		Decreased survival, decreased redox and DNA repair, stimulated apoptosis	[104]
miR-328-3p	Treatment with miR-328-3p	HOS-2R, U2OS + HOS xenograft mice	Decreased survival, increased apoptosis, decreased DNA repair	[105]
iNOS, Nitric Oxide Synthase	Plasmid iNOS	D17 canine OS cell line	Decreased cell survival under hypoxic conditions	[106]
UBE2T, Fanconi anemia gene, ubiquitine ligase	shRNA	U-2OS MG63, xenograft	Decreased survival fraction, induced cell cycle arrest in G2/M, promote apoptosis	[107]
AKT2, serin/threonin kinase	miR-203a-3p	MG-63	Promoted apoptosis	[108]
IGF1R, Insulin-Growth Factor Rceptor	siRNA	U2, MG63, LM-8, SaOS-2, murine xenograft model	Suppressed growth, arrested cells in G0/G1, induced apoptosis, increased cell death,	[109]
**Ewing Sarcoma**				
Survivin, anti-apoptotic protein	SiRNA	4 EWS cell lines RM-82, CADO-ES-1, VH-64, STA-ET-1	Increased number of radiation-induced DSBs, reduced repair, increased apoptosis, reduced proliferation	[95]
**Chordoma**				
RAD51, recombinase	shRNA	U-CH1, U-CH2	Decreased cell viability, increased apoptosis	[69]

Future directions could also lead to the combination of immunomodulators and radiotherapy. It is now widely accepted that RT can trigger a systemic immune response supporting a strong rationale for the combination of RT and immunotherapy [110]. Radiations induce a series of biological effects including enhancing tumor antigen release and presentation, promoting priming and activation of immune cells, increasing density of tumor-infiltrating lymphocytes, facilitating recognition of tumor cells by T cells [110]. Combination of immunotherapy and radiotherapy has been evaluated in different solid tumors including melanoma, Non-Small Cell Lung Cancer and other solid tumors. The efficiency of Immune Checkpoint Inhibitors as single agents in bone sarcoma patients has been limited [111, 112]. Given the strong systemic anti-tumor immune effect induced by radiotherapy, an interesting rationale could be the combination of radiotherapy and immune checkpoint inhibitors. To our knowledge, no study has been reported in bone sarcoma concerning radiotherapy-induced anti-tumor immunity, or proof of concept of the combination of radiotherapy and immunotherapy so it would be crucial to investigate further pre-clinically the rationale and to determine efficient and precise biomarkers to predict and evaluate response to this kind of treatment.

### Combination of radiotherapy and pharmacological/genetic inhibition of targets in bone sarcoma in clinical trials

Ongoing clinical trials combining drugs with radiotherapy are summarized in Table 9. In CD, 2 clinical trials show promising combinations. These trials evaluated the efficacy of a combination of an anti-brachyury vaccine with radiotherapy. Brachyury is involved in CD tumorigenesis, progression and poor prognosis, and the vaccine targeting brachyury as monotherapy is in phase I. The results of the phase I clinical trial of brachyury vaccine as monotherapy have demonstrated that brachyury vaccine induces a specific immune response. As radiotherapy can induce immunogenic cell death triggering a strong immune response, the combination of brachyury vaccine and radiotherapy could have a synergistic effect. Other studies combining different chemotherapy regimens with radiotherapy are being tested in OS and EWS.

**Table 9:** Clinical trials combining radiotherapy and FDA approved drugs in bone sarcoma

Clinical trials	Patients included	Drug	Radiation	Phase	Status	Evidence level
NCT03595228	29 avanced CD	BN-Brachyury	Fractionated radiation	2	Active, Not recruiting	1c
NCT01407198	29 advanced CD	Nilotinib (BCR-Abl, c-kit, and PDGF)	Fractionated radiation	1	Active, not recruiting	1c
NCT02383498	55 advanced CD	GI-6301 brachyury vaccine	70 Gy fractionated radiation	2	Unknown	1b
NCT02802969	64 advanced CD after incomplete surgery	Hypoxia: 18F FAZA, proton boost	Proton therapy	2	Recruiting	1c
NCT02989636	33 recurrent, advanced or metastatic CD	Nivolumab (anti PD-1 antibody)	Stereotactic radiosurgery	1	Recruiting	1c
NCT01696669	43 EWSs	Chemotherapy: vincristine, doxorubicine, ifosfamide-etoposide, dexrazoxane-cyclophosphamide	Radiotherapy after incomplete resection	2	Completed	1c
NCT00023998	80 metastatic OSs	Trastuzumab (HER2)	radiotherapy	2	Completed	1c
NCT01886105	4 metastatic OSs	Sm-EDTMP	Radiotherapy	2	Terminated	1c
NCT03612466	20 OSs bone metastases	153Sm-DOMTP Calcium carbonate Mozobil Neupogen	Radiotherapy	1	Not yet recruiting	1c
NCT00002466	Bone sarcoma	Cyclophosphamide, doxorubicin hydrochloride, etoposide, ifosfamide, vincristine sulfate, surgery	Radiotherapy	2	Completed	1c
NCT00245011	11 OSs	Samarium-153	Radiation	2	Completed	1c
NCT00544778	7 recurrent bone sarcomas	Filgrastim, dexrazoxane, doxorubicin, ifosfamide, irinotecan, conventional surgery	Radiotherapy	2	Terminated	1c
NCT03539172	61 bone sarcomas of head and neck	Apatinib mesylate	radiotherapy	2	Unknown	1c
NCT04398095	20 radiation-induced bone sarcomas	Hyperthermia	Radiotherapy	2	Recruiting	1c

Other studies are necessary to test the efficacy of specific targeted therapy that could theoretically play a role in the response to radiotherapy. With the development of new radiotherapeutic approaches and their improved efficacy, specific studies deciphering the mechanistic action of these approaches in bone sarcoma would be not only interesting, but welcome to gain further insight into personalized medicine.

### Toxicity & limitations

The improved efficacy of new radiotherapy techniques, such as proton beam or carbon ion therapy, offers new therapeutic perspectives in bone sarcoma. However, radiotherapy is still associated with short- and long-term toxicity, as described in Tables 2 and 4. Toxicity depends on the location of the tumor, and children are often particularly vulnerable to radiation-induced late toxicity and to secondary malignancies due to their immature tissue. In a cohort of 222 patients (151 skull-base CD and 71 CHS) treated post-operatively with proton therapy, long-term high grade (> 3) toxicity-free survival was 87%. High-grade late toxicity was characterized by optic neuropathy, temporal lobe necrosis with cerebellum brain parenchyma Grade 3 necrosis, spinal cord necrosis and unilateral hearing loss [113]. In spinal tumors, spinal cord toxicity and insufficiency fractures are the most common radiotherapy-associated side-effects observed [114]. In children pelvic Ewing sarcoma, radiation can cause pelvic pain, premature ovarian deficiency, unequal limb length due to slow bone growth [115]. Aside from radiotherapy toxicity, one major drawback in cancer patient treatment by radiotherapy is the cost and lack of accessibility with only 30 proton therapy centers in Europe.

## Conclusion

Bone sarcomas are a group of rare and heterogenous tumors, affecting people of all ages. Surgery is still the mainstay of bone sarcoma patients’ treatment. However, due to the localization of the tumor and the co-morbidity associated with surgery, complete resection is often difficult. Radiotherapy is used in case of incomplete resection or for unresectable tumors.

In the last decades, there has been an improvement in radiotherapy, both in terms of methods of delivery and types of radiation used, leading to more important doses delivered to tumors and less toxicity for surrounding healthy tissue. Currently, retrospective cohorts, case–control studies and systematic reviews are the main studies evaluating the efficacy of radiotherapy in bone sarcoma. Thus high-quality, multicentric randomized controlled trials are desperately needed to precisely determine the benefits of radiotherapy in bone sarcoma. Efforts are ongoing to standardize the treatment in these rare diseases, regroup patients into adapted clinical trials, and improve patient management. A better understanding of the cellular and molecular mechanisms induced by radiotherapy could offer new therapeutic perspectives.

In vitro and in vivo pre-clinical data combining drugs and radiotherapy have shown promising results in bone sarcomas. However, it is important to remember that during the last decade, very few new drugs have been approved for concurrent radiotherapy administration in other cancers where pre-clinical data were also promising. Out of hundreds of clinical trials, only 2 compounds were finally approved for concurrent radiotherapy: the alkylating agent temozolomide and the anti-EGFR antibody cetuximab [116]. This highlights clear gaps between experimental models and the clinical reality that need to be addressed in bone sarcoma research. Efforts need to be made to improve translational research through in vitro and in vivo models to match radiotherapy specificities and challenges, but also through experimental design revision to unveil synergistic combinations. This need is particularly illustrated by the most recent studies showing the strong efficiency of immunotherapy combined to radiotherapy, even in immune desert tumors [117]. The tumor microenvironment plays a primordial role in tumor initiation and progression and a way to improve tumor modeling could be to reproduce the TME, both in vitro and in vivo. This could be of particular interest in CHS and CD, which are considered immune desert tumors, and where radiotherapy could reverse tumor immune desertification. Finally, strategies focusing on the delivery of targeted therapies and radiotherapy may also offer improved approaches in the treatment of bone sarcoma.

## Data Availability

Non applicable.

## Abbreviations

OS: Osteosarcoma
EWS: Ewing Sarcoma
CHS: Chondrosarcoma
CD: Chordoma
TP53: Tumor protein 53
FLI1: Friend Leukemia Integration 1
IDH: Isocitrate DeHydrogenase
RT: Radiotherapy
IMRT: Intensity Modulated RadioTherapy
SBRT: Stereotactic Body Radiation Therapy
ROS: Reactive Oxygen Species
ATM: Ataxia Telengiectasie Mutated
ATR: Ataxia Telengiectasie RAD3-related
H2AX: H2A.X variant histone
CHK: Checkpoint kinases
MCM: MiniChromosome Maintenance
RPA: Replication Protein A
PCNA: Proliferating Cell Nuclear Antigen
MDM2: Murine Double Minute 2
CDKN2A: Cyclin Dependant Kinase Inhibitor 2A
CDK4/6: Cyclin Dependant Kinase 4 and 6
Rb1: Retinoblastoma protein 1
E2F: E2 factor
RAD: Recombinase
NBS1: Nijmegen Breakage Syndrome 1
CRIF: CR6 Interacting Factor
53BP1: P53 binding protein 1
PRKDC: Protein Kinase DNA-activated, Catalytic sub unit
LIG4: DNA ligase 4
XRCC4: X-Ray Repair Cross Complementing 4
XLF: XRCC4 Like Factor
CTIP: CtBP Interacting Protein
PARP-1: Poly ADP ribose polymerase
Bcl proteins: B cell lymphoma proteins
XIAP: X-linked inhibitor of apoptosis
TNFSFR: Tumor Necrosis Factor Superfamily Receptor
PLK1: Polo Like Kinase 1
WEE1: G2 checkpoint kinase
PI3K: Phosphoinositide 3 phosphate
HDAC: Histone DeACetylase
iNOS: Nitric Oxide Synthase
UBE2T: Ubiquitin-conjugating enzyme E2 T
Akt: Protein kinase B
IGF2R: Insulin Growth Factor Receptor
